# Comparison of multileaf collimator and conventional circular collimator systems in Cyberknife stereotactic radiotherapy

**DOI:** 10.1093/jrr/rrw130

**Published:** 2017-02-13

**Authors:** Taro Murai, Yukiko Hattori, Chikao Sugie, Hiromitsu Iwata, Michio Iwabuchi, Yuta Shibamoto

**Affiliations:** 1 Department of Radiology, Nagoya City University Graduate School of Medical Sciences, 1 Kawasumi, Mizuho-cho, Mizuho-ku, Nagoya 467-8601, Japan; 2 Department of Radiation Oncology, Nagoya Proton Therapy Center, Nagoya City West Medical Center, Nagoya, Japan; 3 Yokohama CyberKnife Center, Yokohama, Japan

**Keywords:** multileaf collimator, circular collimator, cyberknife, prostate cancer, liver cancer, intracranial tumor

## Abstract

Multileaf collimator (MLC) technology has been newly introduced with the Cyberknife system. This study investigated the advantages of this system compared with the conventional circular collimator (CC) system. Dosimetric comparisons of MLC and CC plans were carried out. First, to investigate suitable target sizes for the MLC mode, MLC and CC plans were generated using computed tomography (CT) images from 5 patients for 1, 3, 5 and 7 cm diameter targets. Second, MLC and CC plans were compared in 10 patients, each with liver and prostate targets. For brain targets, doses to the brain could be spared in MLC plans better than in CC plans (*P* ≤ 0.02). The MLC mode also achieved more uniform dose delivery to the targets. The conformity index in MLC plans was stable, irrespective of the target size (*P* = 0.5). For patients with liver tumors, the MLC mode achieved higher target coverage than the CC mode (*P* = 0.04). For prostate tumors, doses to the rectum and the conformity index were lowered in MLC plans compared with in CC plans (*P* ≤ 0.04). In all target plans, treatment times in MLC plans were shorter than those in CC plans (*P* < 0.001). The newly introduced MLC technology can reduce treatment time and provide favorable or comparable dose distribution for 1–7 cm targets. In particular, the MLC mode has dosimetric advantage for targets near organs at risk. Therefore, the MLC mode is recommended as the first option in stereotactic body radiotherapy.

## INTRODUCTION

The CyberKnife® (Accuray Inc., Sunnyvale, USA) is an image-guided, frameless robotic radiotherapy system. The system consists of a 6 MV linear accelerator mounted on a robotic arm that is able to deliver radiation from hundreds of automated positions spaced uniformly around the target [1–3]. The beam size is controlled through either a fixed circular collimator (CC) system with the choice of 10 circular beams of diameters ranging from 0.5 to 6 cm. In a typical conventional CC plan, hundreds of non-isocentric and non-coplanar circular radiation beams are pointed to the edge of the target, creating a highly conformal dose distribution with sharp dose drop-off at its periphery and a low dose to adjacent organs at risk. These characteristics make the Cyberknife system ideal for treatments that require high spatial accuracy and high conformity such as stereotactic radiotherapy and intensity-modulated radiotherapy. However, the conventional CC system requires a large number of monitor units to deliver the desired dose. Clinically, using the conventional CC system, irradiation usually requires 30–60 min in addition to 5–10 min of set-up, so only a limited number of patients can be treated with the conventional CC system in one day. Tumor motion during treatment can also be problematic. In addition, the dose to the target is non-homogeneous, with prescription isodose lines typically ranging between 50% and 85% of the maximum dose. Based on these reasons, large targets are not considered as candidates for conventional Cyberknife treatment [1].

Recently, multileaf collimator (MLC) technology has been introduced for the Cyberknife [4, 5]. The new MLC system, named InCise^TM^, consists of 41 leaf pairs, each with a width of 2.5 mm. The maximum field size is 12 cm × 10.25 cm. This new system allows the fields to be shaped freely to match the tumor shape. The MLC system can be expected to enable more efficient dose delivery and to provide better dose distributions, especially for the treatment of large targets. In addition, the fields can be shaped to spare critical organs in the MLC system. Thus, targets near critical organs may also be good candidates for the new system. However, target size, target location and overlap with critical organs may affect the dose distribution in the Cyberknife system, and the relationship among them has not been clarified. While the new MLC system has an advantage in reducing treatment time, the clinical indication of the MLC system has not been established yet. Therefore, we conducted a planning study to compare CC and MLC treatment plans. First, to clarify the relationship between tumor size and treatment systems, CC and MLC plans were generated for brain targets of various sizes. Second, dosimetric comparisons were carried out in actual patients with prostate or liver tumors. To our knowledge, this is the first study that has evaluated the relationship between target size and locations of tumor and critical organs and a clinical indication of the new system.

## METHODS

### Study design

Dose distributions and treatment times in CC and MLC plans were compared in three clinical situations: (i) brain target volume, (ii) liver target volume and (iii) prostate target volume. First, to investigate suitable target sizes for MLC plans, spherical planning target volumes (PTVs) with diameters of 1, 3, 5 or 7 cm were contoured on the CT images of 5 patients. In total, 40 plans were analyzed in this step. To evaluate the interaction between treatment mode and target size, multivariate analysis was carried out. As a second step, to demonstrate the advantages of the MLC system in actual stereotactic body radiotherapy, MLC and CC plans were generated for 10 patients with liver tumors and 10 patients with prostate tumors. Parameters in both plans were evaluated. These imaging data were obtained from those of patients treated at Nagoya Proton Therapy Center.

### Ethics approval and consent to participate

This study was reviewed and approved by the institutional review board (No. 60160054).

### Planning

The patients were placed in a supine position on a vacuum bag or a headrest with a malleable thermoplastic mask molded to the patient's head. Thin-sliced high-resolution CT images (1.25 mm for the brain targets and 2.5 mm for the prostate and liver targets) were obtained through the region of interest. Contrast-enhanced CT images were taken and fused onto unenhanced images. The prescription dose was defined as the dose covering 95% of the PTV. To compare dose distributions in these plans, the minimum and maximum doses of the PTVs, dose distribution in organs at risk, treatment time, and monitor unit were evaluated in the planning system. Conformity index (CI) and homogeneity index (HI) were calculated according to the following formulae [6, 7]:

(1) Homogeneityindex(HI)=MaximumdosePrescribeddose

(2) $$\mathrm{Conformity}\;\mathrm{index}\;(CI)=(V_{PTV}/TV_{PV})/(TV_{PV}/V_{TV}).$$

In these formulae, the abbreviations are: *V*_*PTV*_ = PTV (cm^3^), *TV*_*PV*_ = lesion volume (cm^3^) covered by the prescribed isodose, and *V*_*TV*_ = prescribed isodose volume (cm^3^). Lower CI indicates higher conformity, and lower HI indicates better homogeneity. The ideal CI and HI values are both 1.

All contouring and dose planning was performed on unenhanced CT images with Multiplan treatment planning system ver. 5.1 (Accuray Inc., Sunnyvale, USA). The finite size pencil beam and Ray-Tracing were applied as dose calculation algorithms [5]. Inverse optimization was applied to all plans. In the optimization, the same parameter sets were used in both MLC and CC plans for the same target to reduce uncertainty in the planning process. All optimization procedures were carried out until breaking of the PTV dose constraints or satisfying other organs dose constraints. When the PTV constraints were broken, the optimization was restarted over again. In CC plans, two collimators were selected automatically among 0.5, 0.75, 1, 1.25, 1.5, 2, 3, 4, 5 and 6 cm collimators. The MLC plans generated with MLCs allow a full body path using non-isocentric beams with 2 mm leaf margins. In cases with targets near critical organs, the MLC was shaped to reduce the doses to these organs.

### Structure definitions and dose constraints

In brain plans, spherical PTVs were contoured in the center of the brain. The prescribed dose was 30 Gy in three fractions. To avoid dose spills, two ring structures (shells) were placed within a radius of 3–15 mm from the PTV. Treatment time, HI and CI for the PTVs, and V20 Gy, V10 Gy and V5 Gy for the brain were evaluated. The V*x*Gy value represents the percentage volume (cm^3^) receiving *x* Gy. In the liver tumor plans, the visible enhanced lesion was contoured as the clinical target volume (CTV). The CTV was expanded by 5 mm for the PTV, and 55 Gy/10 fractions was prescribed to the PTV. Organs at risk included the liver, small intestine, colon, stomach and spinal cord. The maximum doses to the small intestine, colon, stomach and spinal cord were set at <40 Gy, 45 Gy, 45 Gy and 37 Gy, respectively. The V30 Gy of the liver was set at <25% of the liver volume. In the prostate plans, all patients had Stage I–II prostate cancer according to the 7th edition of TNM staging at clinical diagnosis. Thus, the prostate was contoured as the CTV in accordance with a previous report [8]. The CTV was expanded by 3–5 mm for the PTV (5 mm in the lateral, cranial–caudal and anterior directions and 3 mm in the posterior direction). The prescribed dose was 36.5 Gy in 5 fractions. As organs at risk, the rectum and bladder were contoured on non-contrast CT. Dose constraints were: (i) rectum: V50% < 50%, (i.e. the volume receiving 50% of the prescribed dose was <50%), V80% < 20%, V90% < 10% and V100% < 5%; and (ii) bladder: V50% < 40% and V100% < 10%.

### Statistical analyses

Comparisons of dose–volume parameters and treatment time between CC and MLC plans were carried out using the Mann–Whitney test for univariate analysis. In the brain plan analyses, two-way analysis of variance (ANOVA) was carried out in the multivariate analyses. In the analyses, the effect of other variables can be removed and the interaction effect of two variables can be calculated. All statistical analyses were performed using R version 3.0 (the R Foundation for Statistical Computing, Vienna, Austria). Statistical significance was defined as *P* ≤ 0.05. All planning and evaluation was performed by a single radiation oncologist (T.M.).

## RESULTS

**Fig. 1.**
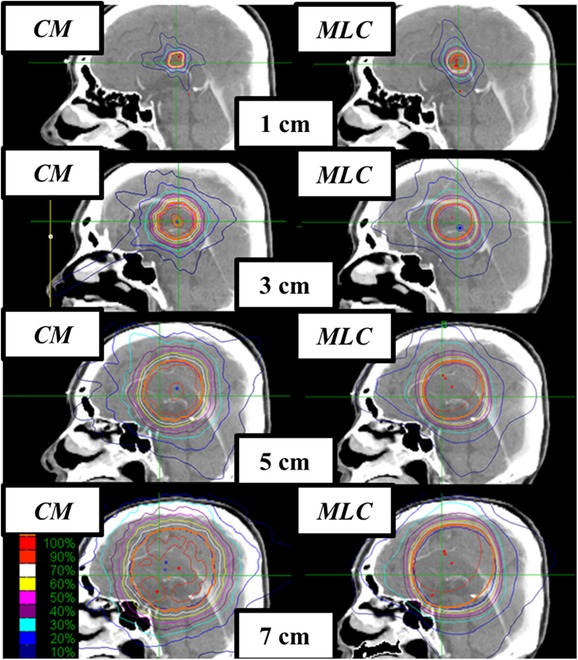
Dose distribution for brain target volumes of 1, 3, 5 and 7 cm maximum diameter. Orange line indicates prescribed dose.

**Fig. 2.**
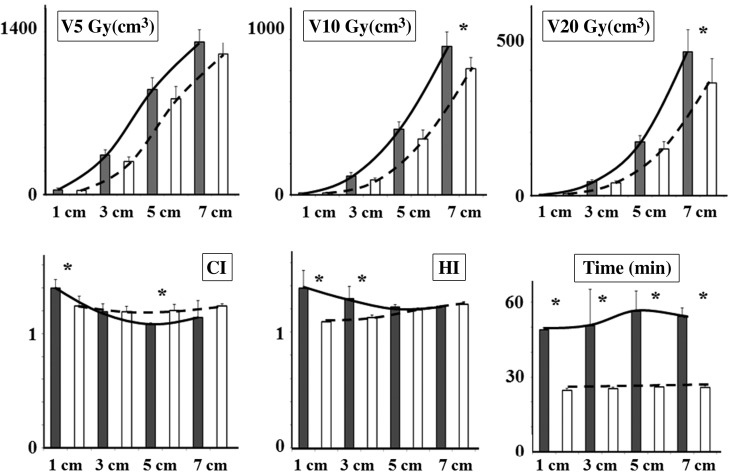
Dose–volume parameters in circular collimator and multileaf collimator plans for 1–7 cm brain target volumes. ^*^Significant difference in univariate analyses (*P* ≤ 0.05). White bars indicate multileaf collimator and gray indicate circular collimator.

The typical dose distributions in brain plans are shown in Fig. 1. In CC plans, except for one case, patients with 1 cm targets were treated with 0.5 and 0.75 cm CCs, 3 cm targets with 1.25 and 2 cm CCs, 5 cm targets with 2 and 3 cm CCs, and 7 cm targets with 3 and 4 cm CCs. Combination of 1.25 and 1.5 cm CCs were applied to one case with a 3 cm target. The results of the multivariate analyses are summarized in Table 1 and Fig. 2. In two-way ANOVA, the effect of each variable was calculated to remove the effects of other variables (Table 1). The multivariate analyses showed that the treatment system was a significant factor regarding HI and maximum and minimum doses (Table 1, fourth column). The minimum dose coverage in MLC plans was higher (28.6 ± 0.6 Gy vs 26.8 ± 1.6 Gy, *P* < 0.0001) and the HI was lower (1.16 ± 0.06 vs 1.28 ± 0.10 Gy, *P* < 0.0001) (Table 1, fourth column). The MLC mode could achieve more uniform dose delivery. However, the differences became smaller as the target size became larger for the interaction effects between the treatment system and tumor size (Fig. 2, HI, *P* < 0.0005). In the univariate analyses, for the 1 cm and 3 cm targets, the MLC plans achieved more homogeneous dose delivery to the targets than the CC plans (Fig. 2, HI, *P* ≤ 0.02), whereas no significant differences were observed with 5 cm and 7 cm targets (Fig. 2, HI, *P* = 0.64). Similar results were observed for the minimum and maximum doses of the targets. Besides, multivatiate analyses indicated that target size was a significant factor for CI (Table 1, ninth column, *P* < 0.0001). In the CC plans, CIs differed depending on target size (one–way ANOVA, *P* < 0.001), whereas those in the MLC plans were stable (*P* = 0.35). For 3–7 cm targets, the mean CI in the CC plans was favorable or at least acceptable compared with that in the MLC plans (Fig. 1).

**Table 1.** Multivariate analyses of brain plans

	Treatment system			Target size				
	(Mean ± SD^a^)			(Mean ± SD)				
	MLC^b^	CC^c^	*P*-value	1 cm	3 cm	5 cm	7 cm	*P*-value
V5 Gy (cm^3^)	586 ± 476	650 ± 509	0.01*	39 ± 13	309 ± 51	863 ± 105	1260 ± 110	<0.0001*
V10 Gy (cm^3^)	320 ± 323	377 ± 380	<0.001*	11 ± 3	106 ± 22	390 ± 60	886 ± 108	<0.0001*
V20 Gy (cm^3^)	138 ± 146	169 ± 186	0.02*	4 ± 1	42 ± 5	159 ± 24	408 ± 88	<0.0001*
CI^d^	1.22 ± 0.06	1.21 ± 0.16	0.88	1.34 ± 0.13	1.19 ± 0.06	1.14 ± 0.07	1.19 ± 0.11	<0.0001*
HI^e^	1.16 ± 0.06	1.28 ± 0.10	<0.0001*	1.23 ± 0.18	1.21 ± 0.11	1.21 ± 0.02	1.23 ± 0.02	0.7
Maximum (Gy)	34.8 ± 1.9	37.5 ± 3.8	0.006*	36.9 ± 5.5	36.3 ± 3.3	36.2 ± 0.7	37.1 ± 0.5	0.54
Minimum (Gy)	28.6 ± 0.6	26.8 ± 1.6	<0.0001*	27.5 ± 2.4	27.6 ± 1.8	28.0 ± 0.5	27.6 ± 0.6	0.8
Time (min)	25.5 ± 1	52.7 ± 8.6	<0.0001*	37.1 ± 13.6	38.0 ± 16.5	41.2 ± 16.9	40.0 ± 15.1	0.46

^a^Standard deviation, ^b^multileaf collimator, ^c^circular collimator, ^d^conformity index, ^e^homogeneity index. *Significant difference in multivariate analyses (*P* ≤ 0.05).

Multivariate analyses indicated that doses to the brain were well spared in the MLC plans. V5 Gy, V10 Gy and V20 Gy were significantly lower with the MLC mode (Table 1, fourth column, *P* ≤ 0.02). As target size increased, doses to the brain increased (Table 1, ninth column, *P* < 0.0001). In addition, the interaction effect between treatment modality and target size was observed in V10 Gy and V20 Gy (Fig. 2, *P* ≤ 0.02). The differences in these parameters widened with a larger target size (Fig. 2, V10 Gy and V20 Gy). In the MLC plans for 7 cm targets, those for the brain could be spared by 15% (Mann–Whitney U test, *P* = 0.03) and 22% (*P* = 0.03), respectively. Although no significant difference was observed in V10 Gy and V20 Gy of the brain for 1–5 cm targets in univariate analyses (*P* ≥ 0.09), these mean parameters were lower in MLC plans than in CC plans (Fig. 2, V10 Gy and V20 Gy). Irrespective of the target diameter, treatment time decreased significantly to 32% in MLC plans compared with in CC plans (Table 1, fourth column, *P* < 0.0001).

**Fig. 3.**
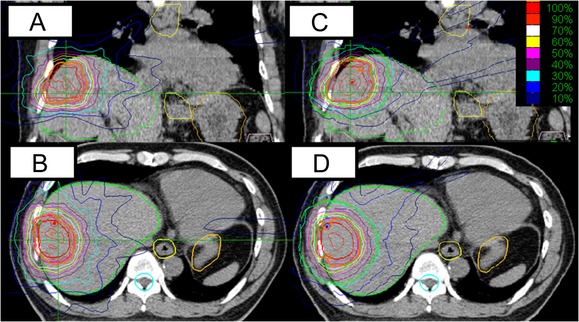
A typical liver target volume plan. (A) Coronal image of the circular collimator plan. (B) Axial image of the circular collimator plan. (C) Coronal image of the multileaf collimator plan. (D) Axial image of the multileaf collimator plan. Orange lines indicate the prescribed dose.

For the liver tumors, the typical dose distribution is shown in Fig. 3. Table 2 summarizes the dosimetric parameters and treatment times of the 20 plans of 10 patients. The median PTV was 51 cm^3^ (range, 26.1–283 cm^3^). The diameter ranged from 2 to 4 cm. Four tumors were located in S8, 2 in S7, 1 in S5, 1 in S4, 1 in S3, and 1 in S1. No overlap volumes between the PTV and critical organs (the colon, small intestine, esophagus, stomach and spinal cord) were observed. The CI, HI and V30, 20, 10 and 5 Gy did not differ significantly in either the MLC or CC plans (*P* ≥ 0.15). On the other hand, the maximum doses to the critical organs were reduced in the MLC plans (Table 2, *P* < 0.05), and the minimum dose coverage was higher in the MLC plans than in the CC plans (51.8 vs 49.3 Gy, *P* = 0.001). Treatment plans with MLC could shorten treatment time (38 vs 61 min, *P* = 0.001).

**Table 2.** Dosimetric comparison of liver plans

		Mean ± standard deviation		*P*-value
		MLC^b^	CC^c^	
PTV^a^	Maximum (Gy)	69.2 ± 2.4	68.7 ± 3.6	0.65
	Minimum (Gy)	51.8 ± 0.8	49.3 ± 1.3	0.001*
	CI^d^	1.39 ± 0.15	1.28 ± 0.25	0.15
	HI^e^	1.26 ± 0.6	1.25 ± 0.07	0.65
	Time (min)	38 ± 4	61 ± 12	0.001*
	Monitor unit	19,159 ± 11,738	61,752 ± 23,544	0.002*
Liver	V5 Gy (%)	49.9 ± 22.5	55.6 ± 22.8	0.54
	V10 Gy (%)	41.4 ± 28.6	36.6 ± 18.5	0.97
	V20 Gy (%)	16.8 ± 10.2	18.2 ± 9.8	0.8
	V30 Gy (%)	9.8 ± 5.3	11.2 ± 6.2	0.74
Bowel^f^	Maximum (Gy)	0.9 ± 1.3	1.2 ± 1.0	0.56
Stomach	Maximum (Gy)	0.8 ± 0.5	1.3 ± 0.4	0.03*
Esophagus	Maximum (Gy)	0.9 ± 0.4	1.3 ± 0.4	0.05*
Spinal cord	Maximum (Gy)	0.5 ± 0.2	0.8 ± 0.4	0.04*

^a^Planning target volume, ^b^multileaf collimator, ^c^circular collimator, ^d^conformity index, ^e^homogeneity index, ^f^colon and small intestine. *Significant difference (*P* ≤ 0.05).

**Fig. 4.**
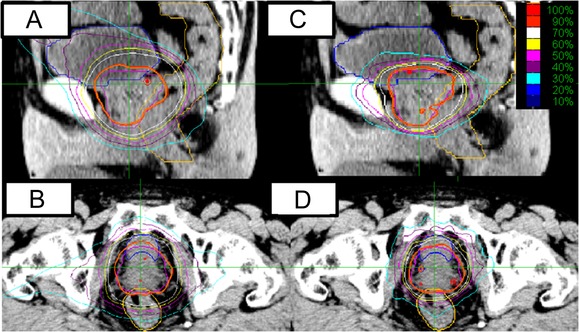
A typical prostate target volume plan. (A) Sagittal image of the circular collimator plan. (B) Coronal image of the circular collimator plan. (C) Sagittal image of the multileaf collimator plan. (D) Coronal image of the multileaf collimator plan. Orange lines indicate the prescribed dose.

In the prostate plans (Fig. 4), the median PTV was 47.5 cm^3^ (range 35–132 cm^3^). The median rectum and bladder volumes were 96 cm^3^ (range, 67–504 cm^3^) and 196 cm^3^ (96–942 cm^3^), respectively. The median overlap volume of the PTV and the rectum was 1.5 cm^3^ (range, 0.6–3.1 cm^3^), while that of the PTV and bladder was 7.0 cm^3^ (4.9–9.6 cm^3^). The maximum and minimum doses and HI did not differ significantly between the MLC and CC modes, but the CI in MLC plans was superior to that in CC plans (Table 3, 1.25 ± 0.03 vs 1.29 ± 0.07, *P* = 0.04). The V50%, V80% and V90% of the rectum in MLC plans were smaller than those in CC plans (*P* ≤ 0.04). The maximum dose to the rectum was lowered by 0.8 Gy in MLC plans compared with in CC plans (*P* = 0.002). Treatment time was reduced in MLC plans (25 vs 31 min, *P* = 0.002). The bladder doses did not differ significantly between MLC and CC plans (*P* ≥ 0.19).

**Table 3.** Dosimetric comparison of prostate plans

		Mean ± standard deviation		*P*-value
		MLC^b^	CC^c^	
PTV^a^	Maximum (Gy)	40.4 ± 0.4	40.8 ± 0.7	0.23
	Minimum (Gy)	34.8 ± 1	34 ± 1.9	0.39
	CI^d^	1.25 ± 0.03	1.29 ± 0.07	0.04*
	HI^e^	1.11 ± 0.004	1.12 ± 0.03	0.63
	Time (min)	25 ± 3	31 ± 5	0.003*
	Monitor unit	20,860 ± 3974	20,772 ± 7084	0.63
Rectum (%)	V50%	18.4 ± 7.5	26.5 ± 8.4	0.02*
	V80%	7.7 ± 3.4	12.3 ± 4.2	0.01*
	V90%	5.6 ± 2.6	8.2 ± 3	0.04*
	V100%	2.4 ± 1	2.7 ± 1.3	0.68
	Maximum (Gy)	37.9 ± 0.3	38.7 ± 0.6	0.002*
Bladder (%)	V50%	29.7 ± 17.9	42.3 ± 19.4	0.19
	V100%	7.2 ± 4.1	8.2 ± 4.6	0.5
	Maximum (Gy)	39.5 ± 2.1	40.2 ± 0.6	0.38

^a^Planning target volume, ^b^multileaf collimator, ^c^circular collimator, ^d^conformity index, ^e^homogeneity index. *Significant difference (*P* ≤ 0.05).

## DISCUSSION

In 2015 MLC technology was introduced for clinical use. However, the dosimetric advantages and clinical indication of the technology have not been fully clarified to date. Previously, two preliminary studies suggested that the addition of MLCs to the Cyberknife system could reduce both treatment time and the total monitor units [4, 5]. Van de Water *et al*. [4] generated lung plans for theoretical Cyberknife models equipped with MLCs using optimization software developed in-house. However, the software could not be applied to the Cyberknife system clinically. McGuinness *et al*. [5] also conducted a planning study using the treatment planning system supplied for clinical use by the vendor. That study suggested that MLC mode can provide a more homogeneous plan with a shorter treatment time. Target coverage and conformity were comparable with our results. However, the prescribed dose and fractionation numbers were not uniform. Only five patients were evaluated in that study. Thus, further investigation was warranted to clarify the indication of the MLC system. In the present study, unified dose-fractionation schedules were prescribed for a larger number of patients to examine two hypotheses: (i) the MLC system can provide better dose distributions, especially for the treatment of large targets, and (ii) the MLC system has dosimetric advantages in the treatment of targets close to critical organs.

First, to investigate suitable target sizes for MLC treatment, plans were generated and analyzed for brain targets of five different sizes. To simplify the model, critical organs were not assumed. In the present study, except for 1 cm targets, the CC system generated favorable or at least comparable dose conformity compared with the MLC system, though the uniformity worsened. Considering that uniformity and conformity have trade-off relations, these results may be reasonable. On the other hand, both the CI and the HI for 1 cm targets were higher than those in MLC plans. That result might be due to the difficulty of achieving a satisfactory target coverage by targeting small pencil beams to the edge of the target. For 1 cm targets, 0.5 and 0.75 cm CCs were used in the planning. These pencil beams may be too small to generate sufficient dose coverage. On the contrary, MLCs are shaped freely to cover the target, and the system can generate sufficient-sized pencil beams to cover the target. The beam number can be reduced in MLC plans, and the surrounding tissues can be spared more efficiently in MLC plans. Compared with the CC plans, the dose conformity and homogeneity were stable in the MLC plans for cases with 1–7 cm targets. Therefore, unless the priority of conformity is higher than that of any other factors, the MLC mode is recommended.

In the next step, these plans were compared in actual clinical patients. Recently, hypofractionated stereotactic body radiotherapy with the conventional CC system has demonstrated favorable clinical results for localized prostate cancers and liver tumors [3, 9, 10]. Therefore, the dosimetric comparisons were carried out in actual patients with prostate or liver tumors. The patients’ images were obtained from those of patients treated with proton therapy. The liver targets were not close to critical organs. The prostate patients were selected as typical candidates with targets close to critical organs. Although the target shape was more complex than that of a simply spherical target, the MLC mode reduced the total treatment time in both prostate and liver target. The results were consistent with those of previous studies and current results for brain targets. The longer treatment delivery time was associated with the larger number of beams. The beam size in the MLC system is larger than that in the CC system. Due to the difficulty of achieving satisfactory target coverage, a larger beam number is needed in CC plans. The duration of a treatment can be reduced by the application of time-reduction techniques aimed at reducing the number of beams [11]. Although the technique is successful, total treatment times remain long for complex clinical cases because of the inherent limitations of using CCs. For prostate or liver tumors, target motion increases during longer treatment times [12]. The motion increases uncertainty in the dose distribution and leads to worsening of toxicity to the critical organs. Therefore, shorter treatment time is clearly an advantage of the MLC system.

In patients with liver tumors, the minimum dose coverage in MLC plans was significantly better than that in CC plans. These results were reasonable and consistent with our brain planning study. On the contrary, the CI was not significantly different. This is apparently incompatible with our results for brain tumors. However, this can be explained by the size of the liver tumors. As described above, compared with the CC plan, doses to the surrounding normal tissues were reduced in MLC plans for larger target sizes. In our experience with brain planning, no significant difference was observed in cases with 3 cm targets (Fig. 2, CI). In the current liver data, the median liver target size was 51 cm^3^ and the diameter was 2–4 cm. The target shape was more complex than that in brain experience. Thus, these may be the reasons that we were unable to detect differences between the MLC and CC plans in the liver study. The V5 Gy, V10 Gy, V20 Gy and V30 Gy in MLC plans did not differ significantly from those in the CC plans. The critical organ doses were also reduced in the MLC plans. According to previous reports, the maximum tumor size ranges from 2 to 5 cm in extracranial stereotactic radiotherapy [1, 13–16]. Therefore, the CI in MLC plans can be comparable with that in CC plans in actual clinical settings. Considering these observations, the MLC mode may be expected to be the first choice in stereotactic body radiotherapy.

Furthermore, the current prostate study suggests that the MLC mode can reduce the dose to the rectum and achieve similar target dose coverage and better conformity. Although the prostate target volumes were similar to the liver target volumes (47.5 cm^3^ vs 51 cm^3^), the CI in the MLC plans was lower than that in the CC plans for the prostate targets. The difference may result from the overlap volume of the target and critical organs. The MLCs were shaped to reduce critical organ doses in the MLC mode, and the MLC mode enabled easier sparing of the adjacent critical organs than the CC mode. In CC plans, reduction of nearby critical organ doses leads to worsening of conformity. Therefore, MLC treatment is considered advantageous in cases with overlaps with critical organs.

## CONCLUSION

The newly introduced MLC technology can reduce treatment time and provide comparable dose distribution for 1–7 cm targets. The MLC mode was especially observed to have a dosimetric advantage for targets near organs at risk. Therefore, the MLC mode should be the first option in stereotactic body radiotherapy.
